# Role of Structural Changes at Vitrification and Glass–Liquid Transition

**DOI:** 10.3390/ma18163886

**Published:** 2025-08-19

**Authors:** Michael I. Ojovan, Dmitri V. Louzguine-Luzgin

**Affiliations:** 1 School of Chemical, Materials and Biological Engineering, The University of Sheffield, Sheffield S1 3JD, UK; 2 Advanced Institute for Materials Research (WPI-AIMR), Tohoku University, Sendai 980-8577, Japan; dml@wpi-aimr.tohoku.ac.jp

**Keywords:** glass, melt, glass transition, configuron, Anderson localization, percolation, Hausdorff–Besicovitch dimension

## Abstract

Structural rearrangements at calorimetric glass transition are behind drastic changes of material characteristics, causing differences between glasses and melts. Structural description of materials includes both species (atoms, molecules) and connecting bonds, which are directly affected by changing conditions such as the increase of temperature. At and above the glass transition a macroscopic percolation cluster made up of configurons (broken bonds) is formed, an account of which enables unambiguous structural differentiation of glasses from melts. Connection of transition caused by configuron percolation is also discussed in relation to the Noether theorem, Anderson localisation, and melting criteria of condensed matter.

## 1. Introduction

While the calorimetric glass transition in amorphous materials is an obvious effect mechanically expressed by the solid-like behaviour, such as the brittleness of glasses, against the liquid-like behaviour, including the plasticity of the molten state, the underlying microscopic, atomic-size mechanisms and structural rearrangements responsible for the transition itself are still poorly understood. This resulted in widely spread affirmations that there is no structural difference between glasses and liquids and that both glasses and liquids are the same fluid state of matter which differ from each other only by the magnitude of viscosity or relaxation times [1]. Apart from the fact that the viscosity being even used on the logarithmic scale cannot serve as a criterion of glass transition—see e.g., Table 4 of Ref. [2] which shows that the viscosity at the calorimetric glass transition spans over four orders of magnitude from 10^8.8^ to 10^13^ Pa·s—it is now acknowledged that “the treatment of vitrification as a process of continuously breaking ergodicity with entropy loss and a residual entropy tending to zero in the limit of zero absolute temperature is in disagreement with the absolute majority of experimental and theoretical investigations of this process and the nature of the vitreous state”, a conclusion which has been explicitly illustrated by model computations [3].

This overview outlines the importance of distinguishing structural differences of amorphous materials below and above the glass transition temperature and highlights the role of the system in understanding the nature of the transformation of glasses into melts. The focus is on utilisation of configuron percolation theory (CPT), aiming to use it on treating experimental data. The term configuron was introduced by Angell and co-authors, who proposed the congruent bond lattice model with the aim of replacing the set of strongly interacting atoms in the condensed matter with a congruent structure of weakly interacting chemical bonds that is easier to analyse [4,5,6,7]. At temperatures *T* > 0, some of the chemical bonds are broken due to thermal fluctuation: the higher *T*, the more bonds are broken. Each broken bond, along with the associated strain-releasing local adjustment of centres of atomic vibrations, is treated following Angell as an elementary excitation termed a configuron. The notion of configuron is universal, as illustrated by Table 1 (modified after [8]).

The configurons formed do not support atoms/molecules bound to each other. On an increase of either temperature or intensity of radiation, which also effectively breaks chemical bonds [9], the concentration of configurons can become so high that percolation via broken bonds occurs. Kantor and Webman [10] have proved that the rigidity threshold of an elastic percolating network is identical to the percolation threshold. Thus, the formation of the percolation cluster made of configurons results in the full loss of rigidity, which is the transformation of a solid into a liquid and which, in the case of amorphous solids, is treated as a glass–liquid transition. Considering glass–liquid transition as melting of amorphous solids, we formulate the melting criterion of solids as the condition of increase of Hausdorff–Besicovitch dimensionality of the set (*S_B_*) of configurons (broken bonds) from zero to $D_{H}={dim}_{H}\left|S_{B}\right|\geq 2.5$. The constant volume jump of heat capacity at the glass transition then closely follows the equipartition theorem resulting from the change of dimensionality of configurons set from 0 in the glassy phase to *D_H_* in the liquid $\Delta C_{V}\approx {\Delta C}_{V}^{trans}={0.5D}_{H}R$, where *R* is the gas constant.

## 2. Structural Differences Between Glasses and Melts

**Figure 1:**
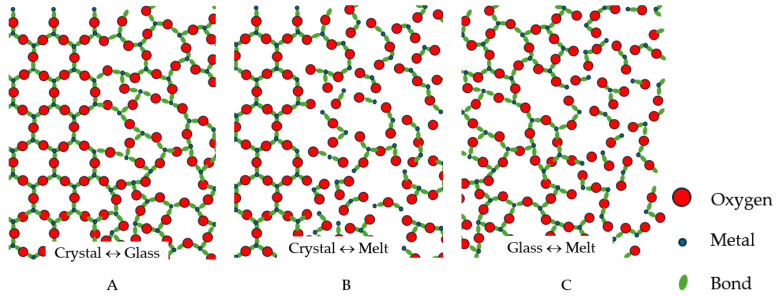
Schematic of interphases between the crystalline, vitreous and molten states of Me_2_O_3_ condensed matter, where Me is a three-valent metal: (**A**): Crystal–glass interface; (**B**) Crystal–melt interface; (**C**): Glass–melt interphase. Both crystals and glasses are fully polymerised practically without any broken bonds at low temperatures. Melts are much less polymerised, containing many finite-sized clusters and many broken bonds compared to solids, where these are not present. Most publications, including recognised handbooks, attribute the same schematic image to both glasses and liquids, erroneously not revealing the large fraction of broken bonds in liquids compared to glasses.

Glasses drastically differ from liquids structurally—thermally disrupted bonds in glasses constitute a small and often negligible fraction of the total number of chemical bonds which provide the integrity and rigidity of condensed matter, while liquids are overloaded by broken bonds. Obviously, the materials become gaseous if all chemical bonds between atoms or molecules are broken, while we expect that they will melt when a significant and well-determined threshold fraction of bonds is broken [11]. Melting is accompanied by a clearly seen change of atomic arrangements in crystalline materials which transform from solid to liquid state via the first-order phase transformation in the Ehrenfest sense, while for glasses the rearrangements of initially disorderedly distributed species (atoms, molecules) are almost undetectable. In the meantime, it is well recognised that the topology of the phase space of glasses drastically changes at the glass transition by reducing its dimensionality [12,13,14,15,16]. One can also state that, topologically, glasses differ from liquids in real space, which is characterised by the set theory via a fractal Hausdorff–Besicovitch (HB) dimensionality (set dimension) *D_H_* equal to ≈ 2.5, while glasses hold an integer *D_H_* which is equal to the dimension of physical space *d* = 3 [8,11,17,18,19,20,21]. The dimension of the set of chemical bonds of materials {*S_B_*} is determined by Minkowski box-covering of the set by boxes with side *ε* via limit:

(1) $${dim}_{H}(S_{B})=D_{H}=\underset{\varepsilon \to 0}{\lim}\frac{\log N(S_{B},\varepsilon )}{\log\left(1/\varepsilon \right)}$$

where *N*($S_{B}$,*ε*) is the number of boxes of the grid intersecting {*S_B_*} [22]. The structural difference between glasses and liquids is hence schematically illustrated by Figure 1.

**Figure 2:**
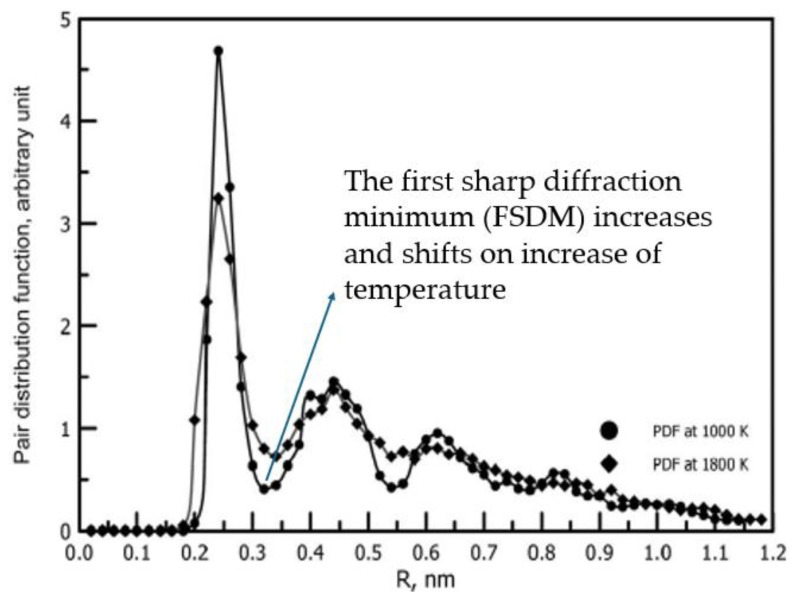
The pair-distribution function PDF of liquid and vitreous Ni with the inset showing a bulk Ni supercell obtained using molecular dynamic simulations (adapted with permission from Ref. [34], AIP Publishing).

This contributed to a misleading belief that glasses having a disordered distribution of atoms similarly to liquids are just liquids but having a very high viscosity, which is arbitrarily set to be higher than about 10^12^ Pa·s, aiming to consider an amorphous material in the glassy state [1,23,24,25,26,27,28,29,30,31,32,33]. The belief that glasses are the same liquids but at high viscosity is amplified by relatively small differences in the X-ray and neutron diffraction patterns exhibited by glasses and liquids, as illustrated by Figure 2, with details provided in [34] (see also Figure 2 of ref. [35]).

In the meantime, not the changes in viscosity but rather the connectivity between species (atoms and molecules) constituting the condensed matter is the governing parameter dictating the state of condensed matter [11,21,36,37,38,39]. Indeed, the viscosity at the calorimetric glass transition can be significantly larger or many orders of magnitude smaller than 10^12^ Pa·s [2,40,41,42], whilst ordered liquid crystals flow at a quite low viscosity, being an ordered state of matter. Table 2 from [39] illustrates this statement by characterising phase states of condensed matter as a function of ordering and connectivity.

**Figure 3:**
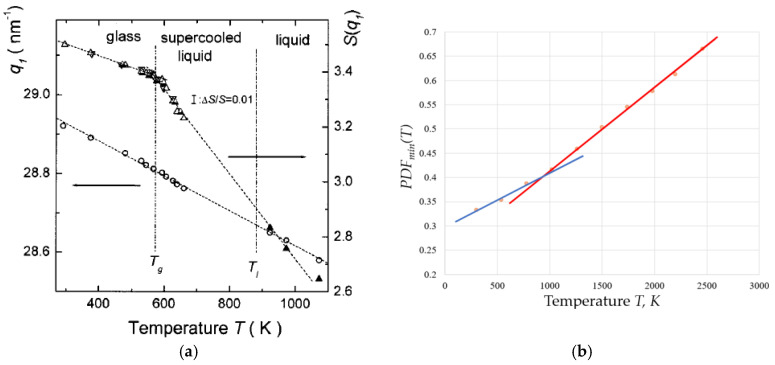
Variation with temperature of structural factor and pair distribution function on crossing the glass transition temperature: (**a**) the first maximum of the structure factor S(q)max and its shifting position q1 reflecting the thermal expansion of Pd40Cu30Ni10P20 bulk metallic glass (reprinted with permission from Ref. [52], AIP Publishing). (**b**) the first sharp diffraction minimum PDFmin of amorphous Ni near Tg = 930 K (adapted with permission from [49]). Copyright 2020 American Chemical Society).

Direct visualisation of the oxide glass structure in line with the existing modified random network model [37] is available [46,47]. Structural differences between glasses and melts were revealed a long time ago, with Wendt and Abraham pioneering the identification of *T_g_* based on them [48]. They have observed the different temperature behaviour of pair distribution functions (*PDF*) *g*(*r*) below and above the glass transition and proposed an empirical (statistically based) criterion for the glass transition by defining the empirical parameter *R_WA_* = *g_min_*/*g_max_*, where *g_min_* and *g_max_* are the magnitudes of the first minimum and first maximum of the *PDF*. It was found that the glass transition caused by changes of temperature (*T*), pressure (*P*), or both *T* and *P* always occurs when *R_WA_* ≈ 0.139–0.142. It was shown later that this threshold coincides with the percolation threshold *R_WA_* = *ϕ_c_* [49] given by the universal Scher–Zallen critical density in the 3D space *ϕ_c_* = *θ_c_* = 0.15 ± 0.01 [50,51]. Among the most important experimental works confirming the structural differences between glasses and melts was the work by Mattern et al. [52], which analysed the thermal behaviour of the structure of Pd_40_Cu_30_Ni_10_P_20_ bulk metallic glass using high-temperature X-ray synchrotron diffraction. The temperature dependence of structure factor *S*(*q*) followed the Debye theory up to the *T_g_,* while above it was altered, indicating structural changes in the liquid. The temperature dependence of structural parameters is different in glass and in supercooled liquid (see Figure 3a), whilst the atomic pair correlation functions *PDF*(*r*) = *g*(*r*) reveals changes in short-range-order parameters of the first and the second neighbourhood with temperature. Figure 3b demonstrates that for amorphous Ni.

Whilst Figure 2 demonstrated the fact that the *PDF*_min_ always increases on an increase of temperature. Figure 3a shows an evident kink of structure factor *S*(*q*) at *T*_g_. Figure 3b demonstrates that the rate of increase of *PDF*_min_ has the same kink at *T*_g_ and becomes higher exactly above it. The changes in the structure of amorphous material at *T*_g_ cause these changes in the macroscopic properties of the material and, first of all, in its thermal expansion—the thermal expansion coefficient of liquid is larger compared to that of glass.

In addition to the well-recognised short-range order (SRO), the medium-range order (MRO) is revealed in both glasses and liquids, where it is emphasised that the atomic pair-distribution function of simple liquids and glasses shows exponentially decaying oscillations beyond the first peak as a representative of MRO [53,54,55,56]. The structural coherence length that characterises the exponential decay freezes at the glass transition and attenuates on increase of temperature [54].

## 3. Role of Configurons in the Phase Transformation

A straightforward description of glass transition as a percolation-type phase transformation between the highly connected glassy state and the less connected liquid phase (see Table 1) is provided by the configuron percolation theory (CPT) [17,18,19,20,21], which can be considered as one of the variants of the well-known two-state model, also referred to as a two-level system, which has been successfully used by glass researchers [57,58,59,60]. The liquid phase is typically treated in the two-state models as a mixture of two types of structural units, with the internal variable being the molar fraction of one or the other type of them, where the CPT uses as units the chemical bonds either intact or broken (configurons—see Table 1), which provide one or another state of matter—see Table 2. Notably, the coupling of the two-state model with well-investigated relaxation models has also been demonstrated in many works [61,62,63,64,65,66,67]. Benigni [57] has accounted for vibrational contributions to the thermodynamic functions using weighted sums of Einstein functions and configurational contributions to the liquid and glass phase functions, applying a single internal variable, the freezing kinetics of which on cooling are calculated with an Adam–Gibbs logarithmic relaxation law [68]. The main conclusions of CPT of glass transition are as follows [69]:

- *Universality*—All disordered systems should exhibit percolation-type transformations from solid-like at higher degrees of connectivity (e.g., at lower temperatures) to fluid-like (plastic) at lower degrees of connectivity (e.g., at higher temperatures or intensity of irradiation).
- *Singularities for derivative parameters*—Thermal expansion, heat capacity, shear modulus, and other properties of glasses show a relatively sudden change at the glass transition temperature. Derivative parameters of amorphous materials thus show typical features of second-order phase transformations, e.g., theoretically, they diverge at *T_g_*.
- *Dimensionality change*—The HB dimensionality of the system of configurons (broken chemical bonds) changes at *T*_g_ from 0 in glasses to fractal *D*_H_ ≈ 2.5 (the experiment shows 2.4–2.8 [69,70,71,72]) for melts.
- *Dynamic (twinkling) fractals*—The glass–liquid transition is accompanied by the formation of a percolation macroscopic cluster made up of broken chemical bonds—configurons. This cluster is similar to Wool’s twinkling fractals [70,71,72]. The percolation cluster is dynamic and changes with time due to configuron migration controlled by diffusion. Nonetheless, at any moment of time there is a percolating cluster made of configurons above the *T_g_*, whereas such macroscopic clusters do not exist in the glassy state (below the *T_g_*). The characteristic linear scale that describes the branch sizes of dynamic clusters formed by configurons is the correlation length *ξ*(*T*);
- *Fractal medium-range order*—The higher the cooling rate, the larger are the remnant fractal clusters frozen at liquid–glass transition. The correlation length gives the average size of clusters made out of broken bonds at *T* < *T*_g_. At *T* > *T*_g_, the correlation length gives the average size of atomic clusters formed. Second-order phase transitions in ordered substances are typically associated with a change in the crystal lattice symmetry, and the symmetry is lower in the ordered phase than in the less ordered phase. In the spirit of Landau’s ideas, the transition from a glass to a liquid spontaneously breaks the symmetry of bonds that is of the system of configurons. At the glass–liquid transition the amorphous material changes the group of isometries from the Euclidean to the fractal space group of isometries at length scales smaller than ξ(*T*).
- *Two activation energies of viscosity*—The viscous flow has a variable activation energy above the glass transition temperature *Q*(*T*), which becomes lower at higher temperatures (Table 3).

**Figure 4:**
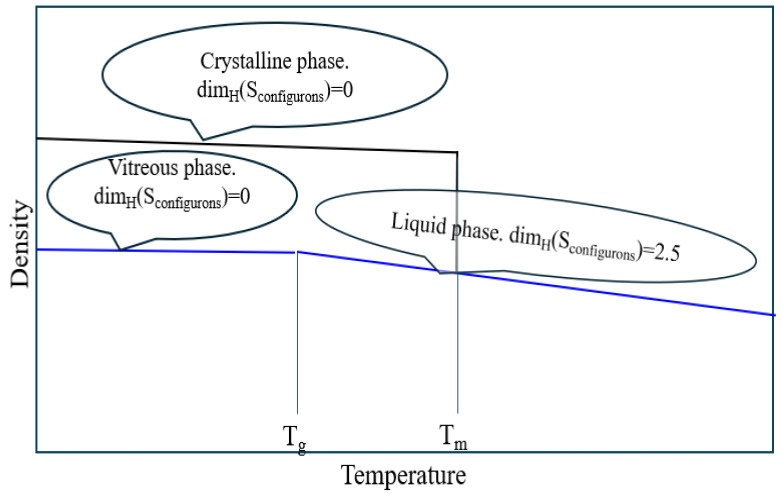
Temperature dependence of density and Hausdorff–Besicovitch (HB) dimensionalities of chemical bonds in materials following CPT.

Fractal structures formed near the glass transition are dynamic structures. On melting, glasses transform to melts, which are supercooled melts above the *T_g_*, and transform to real melts at higher temperatures, i.e., at and above the melting temperature, *T_m_* (Figure 4).

The CPT approach also provides an explicit picture of the melting of solids, enabling the reason for first-order solid-to-liquid phase transformation for crystalline solids and second-order continuous solid-to-liquid transformation for amorphous materials to be revealed [11,69]. The mechanism behind one or another type of melting lies in the mobility of configurons, which is high for crystals because of the periodicity of the crystalline lattice resulting in the equivalence of their positions within the lattice and is low within the disordered lattice of amorphous materials due to the fast localisation of excitations following Anderson’s localisation mechanism [76,77,78,79]. Due to their high mobility in crystals, configurons are quickly migrating to areas of already formed liquid near impurities or surfaces where they condense or partly recombine, adding the heat of condensation and recombination and thus effectively arresting the temperature referred to as the melting point *T_m_*, whereas configurons are highly localised (almost immobile) in amorphous materials, forming geometrically clysters when their concentration becomes high enough without practical release of any heat due to the absence of condensation and recombination processes. In crystals that resemble the boiling process for water when the temperature is arrested at the boiling temperature, with the difference that configurons rather than vapour bubbles are moving through the structure of crystals and are localised in amorphous substances.

Symmetry changes are characteristic for all phase transformations, including the melting of materials, which is the transition from their solid to liquid form. Crystalline materials obey this law with obvious changes of symmetry group from that of a crystal to that of the group of Euclidean isometries of a Euclidean space $E^{n}$, comprising all translations, rotations, and reflections and arbitrary finite combinations of them where n = 3 for 3D space [80]. Symmetry changes are not so obvious for the glass transition, i.e., the transition of amorphous materials from the vitreous to molten state, because both these states belong to the E(3). Symmetry changes become evident for the phase space, which accounts not only for the space location but also for momentum. The breaking of symmetry during phase transitions plays a crucial role in determining the system’s behaviour and the nature of the glass transition [81,82,83]. Within the CPT the main symmetry change is the change of dimensionality of space accessible to configurons from 0 in the glass to the fractal one *D_H_* in the liquid—i.e., here, we observe an increase of dimensionality which can be linked with the new degrees of freedom related to translational motion. Following many publications such as [2,8,9,11,12,13,14,15,16,17,18,19,20,21,36,37,38,39,40,41,42,49,57,69,70,71,72,73,74,84,85], we therefore conclude that the glass transition is a true phase transformation—a specific case within critical phenomena generically termed topological phase transitions, which are amenable to the scaling approach and characterised by diverging length and time at the transition.

## 4. The Jump of Heat Capacity

In the experiment the calorimetric glass transition is always observed as a second-order phase transformation following the Ehrenfest classifications of phase transitions: there is a continuity of material volume and entropy (although with a kink at *T*_g_), and there is a discontinuity of their derivatives at the transition. That specifically allowed the International Union of Pure and Applied Chemistry (IUPAC) to define the glass transition as a second-order transition in which a supercooled melt yields, on cooling, a glassy structure so that below the glass-transition temperature the physical properties vary in a manner similar to those of the crystalline phase [86]. In practice, namely the kinks and discontinuities observed using, e.g., DSC, are used to detect the *T*_g_; hence, most of the data published are those which belong to the so-called calorimetric glass transition [87].

Observing that the melting of substances has only small effects on the volume, cohesive forces, and specific heat, which permitted Frenkel to conclude that “the character of the heat motion in liquid bodies, at least near the crystallization point, remains fundamentally the same as in solid bodies, reducing mainly to small vibrations about certain equilibrium positions” [88]. Moreover, he has also argued that these equilibrium positions are irregular in a liquid, just as in an amorphous solid, but while the equilibrium positions are permanent in a solid, they are not so in a liquid; rather, each liquid atom oscillates for a time about the same equilibrium position, then jumps to a new one [89]. Wallace has refined Frenkel’s qualitative picture of the liquid state of matter by formulating the hypothesis that the liquid contains a universal ion-motional disordering entropy of *Nk_B_∆_W_* relative to the solid, where *k_B_* is the Boltzmann constant and *∆_W_* = 0.80 [90]. He observed from the experiment that, for large-*N* systems, the constant-density entropy of melting contains the universal disordering contribution of *Nk_B_∆_W_,* suggesting that the random structural valleys, which are static structure potentials as sums of harmonic normal modes, are of universal number w^N^, where *ln*(*w*) = *∆_W_* and the experimental estimate for *∆_W_* is 0.80. Thus, the Hamiltonian of the structural valley in materials is the static structure potential, a sum of harmonic normal modes, and an anharmonic correction [91]. Using this approach, he has shown that in quasi-harmonic approximation, the liquid theory for entropy agrees with the experiment at elevated temperatures, to within 1–2% of the total entropy [90,91]. Based on the CPT picture of melting (see below), we conclude that the Wallace parameter *∆_W_* is equal to the HB dimensionality of percolation clusters formed by configurons at melting *D_H_* divided by the dimensionality of space d, i.e., that *∆_W_* = *D_H_*/*d* ≈ 0.8.

The glass transition in amorphous materials is typically revealed using differential scanning calorimetry (DSC), which always reveals a jump of constant pressure and constant volume heat capacity ∆*C*_p_, ∆*C*_v_ at the glass transition temperature *T*_g_ [87]. The appearance of this jump, which is an obvious and generally accepted indication of a phase transformation, is well understood and confirmed as an appearance in the system of new translational degrees of freedom for atomic or molecular constituents of matter [92,93]. We note that always *C*_p_ > *C*_v_ due to the relationship *C_p_* = *C_v_* + *Vα*^2^*B*, where *V* is the molar volume, α is the coefficient of thermal expansion (CTE), and B is the bulk modulus, and thus always *∆C_p_* > *∆C_v_*. Typically one holds *∆C_v_* ≈ 0.85*∆C_p_* [93]. Recent computer experiments by Cockrell and Grimes [94] have unambiguously confirmed that immediately above the glass transition temperature effectively all atoms in inorganic glasses are mobile, while in the glassy state the fraction of mobile atoms is negligible, which stands in line with the CPT of glass transition and its conclusions. They concluded that the atomic mobility is a universal marker of the glass transition which emphasises the role of structural changes resulting in mobilisation of atoms at the glass transition. Moreover, molecular dynamic simulations by [95] have revealed that the jump ∆*C*_p_ of amorphous silica at the glass transition is entirely determined by the component of structural energy.

**Figure 5:**
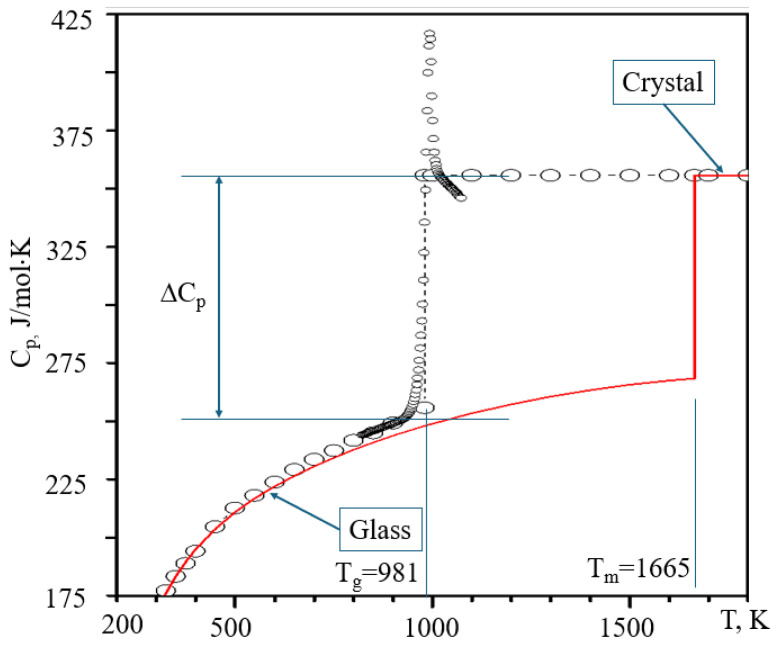
The jump of constant pressure heat capacity at the glass transition of diopside. Courtesy of Reinhard Conrad.

The heat capacity behaviour at glass transition generically has two prominent features: (i) it diverges on the increase of temperature towards *T*_g_ and (ii) has a distinct jump from the lower heat capacity of glass, which is almost the same as that of a crystal, to that of a liquid [96]. Figure 5, modified from [91], shows both these features.

The first feature—the divergence of heat capacity—is given within CPT as a universal law, so on approaching the glass transition temperature, the heat capacity follows the dependence [18,19,20,21]:

(2) $$\Delta C_{V}\propto 1/{\left|T-T_{g}\right|}^{1-\beta }$$

where *β* = 0.41 is the critical index in the 3D space [51]. One can note that experimentally measured critical exponents α for several metallic glasses varied from 0.16 to 0.54 [97], with deviations of α from 1-*β* possibly resulting from a more complex percolating scheme of these metallic systems.

The magnitude of the jump of heat capacity at glass transition is dictated by the liberation of new degrees of freedom, including translational (trans), which can also be related to structural changes, vibrational (vib), and rotational (rot) ones: $\Delta C_{V}={\Delta C}_{V}^{trans}+{\Delta C}_{V}^{vib}+{\Delta C}_{V}^{rot}$, the main component of which is typically the translational one $\Delta C_{V}\approx {\Delta C}_{V}^{trans}$ [92]. In principle, at the transition from one phase (glassy) to another (liquid), some vibrational degrees can be lost; thus, the jump can effectively be diminished, which may be the case for some materials, oxides, and even metallic materials. Within CPT, the constant volume heat capacity jump at the glass transition is directly related to structural changes and to the appearance of new translational degrees of motion and is as follows:

(3) $${\Delta C}_{V}^{trans}=D_{H}R/2$$

Reflecting the equipartition theorem for the change of HB dimensionality of configurons from 0 in the glassy phase to *D_H_* in the liquid (Figure 4). Experimentally it was found that the constant pressure jump of heat capacity at glass transition ∆C_p_ in a variety of metallic glasses is almost an invariable value (13.69 J/mol·K) and is close to 3*R*/2 = 12.47 J/mol·K (where *R* = 8.3145 J/mol·K is the gas constant), which can be quantitatively described by the atomic transitional diffusion [93].

Additionally, it was found that the ratio ∆*C*_v_/∆*C*_p_ does not change with the liquid fragility and almost keeps a constant: 0.85. Hence, the jump of constant volume heat capacity at glass transition of metallic systems is almost constant, ∆*C*_v_ = 0.85·∆*C*_p_ = 11.64 J/mol·K. We observe from CPT that this jump is slightly smaller, namely *D_H_R*/2 = (2.55 ± 0.05)·8.3145/2 = (10.6 ± 0.2) J/mol·K. The constant volume heat capacity jump at glass transition is illustrated in Table 4.

We also note that Table II of reference [92] demonstrated that for most of the substances at glass transition hold 2∆*C_p_*/*R* ≈ 3, the validity of Equation (3) is confirmed because it is known from [93] that *∆C_v_*/*∆C_p_* ≈ 0.85, so that we obtain *∆C_v_*/*∆C_p_* ≈ *D_H_*/*d* in line with (3). Similarly, data from [95] have shown that for amorphous silica the constant pressure jump of heat capacity per atom ranges from 0.50 R to 0.68 R, which means that the jump of constant volume heat capacity is *∆C_v_* ≈ 0.85*∆C_p_* = 10.6–14.4 J/mol·K in line with CPT estimation (10.4–10.8 J/mol·K). It should be noted that Equation (3) is a rough estimation and cannot be universally valid for the overall heat capacity jump at glass transition, having well-known deviations [98,99], and it is only approximately giving the contribution to the overall heat capacity due to liberation at the phase transformation of translational degrees of motion and in this sense is somehow similar in its nature to the Dulong–Petit law. Table 3 demonstrates a deviation of approximately 1 J/mol·K between the calculated and the experimental values, and the source of the errors can be due to the contribution of vibration/rotational degrees of freedom as well as due to changes occurring in the electronic system based on quantum mechanical calculation of electronic density of states (see e.g., Equation (1) of [100]), such as the recently reported localised electronic states which enhance magnetoelectric effects [101]. We emphasise hence that we consider only structural changes related to the calorimetric glass transition, where we have to conclude that no phase transformation occurs in the Ehrenfest sense if neither thermodynamic functions nor their derivatives exhibit any peculiarities.

## 5. Melting Criteria

Melting is defined as a physical process that results in the phase transition of a substance from a solid to a liquid, where the melting point of crystalline solids is the temperature at which a solid changes its state into a liquid at atmospheric pressure, so at the melting point the solid and liquid phases coexist in equilibrium [102]. Encyclopaedia Britannica specifies that amorphous (non-crystalline) substances melt by gradually decreasing in viscosity as temperature is raised without a sharp transition from solid to liquid [103]. We note, however, that melts are much less polymerised compared to glasses, which have a similar structure to that of liquids. Instead of a well-connected network like in solids, they contain many finite-sized clusters as well as many broken bonds, whereas in solids, the latter are almost not present at all or occur as point defects generated by thermal fluctuations (Figure 1) and are characterised by a different dimensionality of the set of configurons (broken chemical bonds).

Lindemann’s and Born’s criteria of melting are the two most frequently used as a basis to analyse the melting conditions [104,105,106]. The Lindemann criterion states that melting occurs because of vibrational instability when the root of mean square vibration amplitude ${\langle u^{2}\rangle }^{1/2}$ exceeds a threshold value taken as a fraction *δ_L_* = (${\langle u^{2}\rangle }^{1/2}/a$) of interatomic distance *a* [96,107,108,109,110]. Lindemann supposed that *δ_L_* should be about 0.5, which was later revised, observing that it is within the range between 0.068 and 0.114 [110]. The analysis of experimental data of elements determined that the Lindemann melting coefficient δ_L_ is in fact an exact value for each element belonging to a given periodic group of Mendeleev’s periodic table of elements [102]. Although it is considered that the Lindemann criterion is supported by data for glass transition, the parameter *δ_L_* is not the same as for the melting of crystals [35,111,112,113]. Finally, we note that Khrapak has shown that Lindemann’s criterion of melting can be formulated for 2D classical solids using statistical mechanics arguments with an expression for the melting temperature derived $(c_{t}^{2}/v_{T}^{2})(1-c_{t}^{2}/c_{t}^{2})≅const$, which is valid for both three and two dimensions [114]. Here, *v_T_* = (*T*/*m*)^1/2^ is the thermal velocity, *c*_l_ is the longitudinal and *c*_t_ is the transverse sound velocity. The expression is reduced to the condition of constant transverse-to-thermal velocity ratio at the melting of the materials, accounting for *c_l_* ≫ *c_t_*.

The Born criterion of melting [105,106] is based on a rigidity catastrophe caused by the vanishing elastic shear modulus so that the crystal spontaneously changes its crystallographic symmetry or becomes fully amorphous, which in many cases can be the melted state, although not necessarily, as amorphisation does not really envisage transition to a molten state. Born’s stability condition is formulated as the condition that $det\left|C^{ijkl}\right|\geq 0,$ where *C^ijkl^* is the (second-order) elastic constant tensor which determines the stress tensor *T^ij^* = *C^ijkl^E^kl^* as a linear function of the infinitesimal strain tensor *E^kl^*. Naturally, for glasses, which are considered isotropic solid materials, the elasticity tensor has only two independent components, which are the bulk (*K*) and shear (*μ*) moduli. We further use an orthonormal Cartesian coordinate basis with no distinction between upper and lower indices. The elastic constant tensor is then written in terms of Lame’s first and second parameters, *λ* and *μ*, correspondingly: *C_ijkl_* = *λδ_ij_δ_kl_* + *μ*(*δ_ik_δ_jl_* + *δ_il_δ_kj_*), where δ_ij_ are the Kronecker’s deltas. The bulk modulus *K =* −*∂p/∂lnV*, where *p* is pressure and *V* is volume, is then *K* = *λ* + 2*μ*/3. The instability of lattices when Born’s criterion is breached does not necessarily cause melting, as it can be due to either a change of lattice symmetry class or amorphisation without melting. It is also worth noting that Born’s criterion was specified for homogeneous lattices under a constant uniform load to be $det\left|B\right|=0,$ where *B* is the four-rank elastic stiffness tensor [115]. Here, we note that instabilities under pressure may also occur for the amorphous state, leading to its phase decomposition; see [116] and the references there.

There is a substantial reason initially outlined by Angell [7] to analyse the distribution and behaviour of broken chemical bonds termed configurons in condensed matter rather than of atoms or electrons since the former are weakly interacting with each other whilst the latter are strongly interacting and form either clusters or are integral parts of the network of material. A configuron is formed by the breaking of a chemical bond, followed by the associated strain-releasing local adjustment of centres of atomic vibration. The Hamiltonian of configurons in the first approximation is that of almost free boson (apart from spin glasses) particles in the periodic (for crystals) or aperiodic (for glasses and liquids) potential created by atoms of material $V(\vec{r)}$ so that the Schrodinger equation for the wave function of a configuron $\varphi_{i}(\vec{r})$ is as follows: $\left(-\frac{\hbar^{2}}{2m}△+V(\vec{r})\right)\varphi_{i}(\vec{r})=i\hbar \frac{\partial \varphi_{i}(\vec{r})}{\partial t}$. In crystalline materials, the potential repeats the symmetry of the lattice; therefore, the wave function of configurons following the Bloch theorem can be represented as $\varphi_{i}(\vec{r})=u_{i}(\vec{r})\cdot exp(i\vec{k}\cdot \vec{r})$, where $u_{i}(\vec{r})$ has the period of the crystal lattice and the exponent is the running wave that carries the momentum $\vec{p}=\hbar \vec{k}$. The configurons are almost freely moving, at least at small wavenumbers k with energy $E=\frac{\hbar^{2}k^{2}}{2m^{*}}$, where m* is the effective mass. The situation for wave propagation drastically changes for disordered lattices amenable to Anderson localisation of configurons instead of almost free motion [75,76,77,78,79]. Indeed, instead of almost free propagation due to identical positions of configurons in the crystal lattice, they quickly localise, which in turn affects the melting process [69]. Once configurons are weakly interacting in the first approximation, they can be considered as almost non-interacting with a random spatial distribution, and for their description, the two-level system can be used, applying the standard apparatus of statistical physics [18,19].

The mutual interaction between bonds and configurons at distances exceeding their sizes, which are approximately equal, can be practically neglected. In this case, the association and formation of clusters of configurons is purely geometrical, depending only on the volume fraction occupied by them, which is well described by the percolation theory [51,117,118,119]. It means that knowing the temperature dependence of the relative concentration of configurons *c* (0 ≤ *c* ≤ 1), one can estimate the probability of cluster formation purely geometrically using c as the occupation probability *p = c*. It is known that *p* plays the same role as the temperature in thermal phase transitions, being the control parameter of the formation of percolation clusters, which are fractal above the percolation threshold *p* > *p_c_* with HB dimension *D_H_* = *d* − *β*/*ν* [51], where *d* is the dimension of space and critical exponents β and ν describe the critical behaviour associated with the percolation transition and are universal, not depending at all on the structure of the lattice and on the type of percolation, which can be either site, bond or even continuum [51,117,118,119]. For *d* = 3 these are approximately as follows: *β* = 0.41, *ν* = 0.88 [51]. The order parameter *P_∞_* of the system describes the probability that a configuron belongs to the percolation cluster [18,19]. Classical percolation exhibits all the characteristics of a continuous phase transition. For *p* ≥ *p_c_,* the order parameter *P_∞_,* which is identified in the CPT as the power of the percolation cluster (fraction of configurons as a part of the largest cluster) made up of configurons, increases with *p* by a power law *P_∞_ ∝ (p* − *p_c_)^β^* (see e.g., Figure 5 of Ref. [19]), while the correlation length describing the inhomogeneities of structure diverges as *ξ ∝ (p* − *p_c_)^−ν^*.

Instead of using the lattice-specific parameter *p,* one can refer to Scher and Zallen [50], who have found that for each dimension there exists an invariant that is almost independent of the type of lattice. This *Invariant ϕ_c_* = *Fpc* is the critical fraction of space occupied by spheres (discs in 2D) of the bond length diameter, positioned in the occupied sites of the lattice. The quantity *f* is called the “filling factor” of the lattice and denotes the volume fraction occupied by mutually touching spheres positioned at each site. The critical space occupation probability equals *ϕ_c_* = 0.44 + 0.02 in two dimensions and *ϕ_c_* = 0.15 ± 0.01 in three dimensions [49,50]. This permits us to calculate the glass transition temperature, *T*_g_, for simple systems such as amorphous silica with only one type of bond (and thus configurons) based on thermodynamic parameters of bonds [17,18,19]:

(4) $$T_{g}=\frac{H_{d}}{S_{d}+Rln(1-\phi_{c})/\phi_{c}}$$

where *H_d_* and *S_d_* are the enthalpy and entropy of connecting bonds (configuron formation), *R* is the universal gas constant, and *ϕ_c_* is the percolation threshold volume invariant approximately equal to 0.15 [44,49,50]. Equation (4) can be used utilising this invariant for simple systems such as amorphous silica, giving for the glass transition temperature *T*_g_ = 1482 K [19] compared with the experimental *T*_g_ value of 1480 K measured by drop calorimetry [120]. Generically, Equation (4) reflects that the more refractory the material, the higher the *T_g_*, including the case of high-entropy metallic glasses, which are characterised by stronger interatomic bonding in the system of different atoms with varying sizes. High-entropy metallic glasses, or glasses without a principal component, sometimes have better mechanical properties than conventional metallic glasses, but finding deep eutectics is more difficult because the multi-component phase diagrams are not available, so the glass-forming ability is reduced [121,122]. Equation (4) can be simplified by neglecting the logarithmic term [11] and reducing it to a view similar to Dienne’s ratio for melting temperatures of substances (presumably crystalline) [123,124], which contains thermodynamic characteristics of joining bonds rather than the enthalpy and entropy of the activated state.

What happens at the transition of condensed matter from a solid to a liquid state, i.e., on melting, e.g., when an amorphous solid (glass) transforms into a liquid? In solids, including glasses, there are both longitudinal and transverse (shear) sound waves, the latter one having two polarisations. Sound waves are characterised by the acoustic dispersion relations *ω_l_(k) = kc_l_*, whereas shear waves have *ω_t_(k) = kc_t_,* where *k* is the wavenumber. These are found from equalities $\rho c_{l}^{2}=K+\frac{4\mu }{3}$ and $\rho c_{t}^{2}=\mu$. In the liquid state the shear modulus becomes nil (below the Frenkel line [125,126,127]); thus, there are only longitudinal modes for sound waves in the molten state. This makes it different from the point of view of stress reaction, although the condition that *μ* = 0 applies only below the Frenkel line, which is at frequencies ω <* ω_F_* = 1/*τ_M_* determined by the Maxwell relaxation time *τ_M_*. At high frequencies, when *ω* > *ω_F_*, all liquids behave solid-like, with both longitudinal and transverse waves propagating with sound velocities given by the same expressions as for solids, where parameters used are the high-frequency adiabatic bulk modulus K_∞_ and shear modulus *μ_∞_*. This well-known property does not result by any means in the conclusion that the liquid state is the same as the solid state of matter. In the meantime, the disappearance of transverse waves means a topological change in the phase space, which is supported by many findings [11,12,13,14,15,16,17,18,19,128]. Loss of transverse momentum signals that one of the symmetries of amorphous materials below the Frenkel line is broken at *T_g_*. A symmetry is a property which will remain the same even after some kind of transformation is applied. Among known symmetries, the dimensionality of the bonding system which provides the condensed character (either solid or liquid) of matter unambiguously changes on passing through the glass transition temperature [21]: the broken bonds of materials (termed configurons [7]) are point-like entities (defects of the glass network) below the *T*_g_, whereas above the glass transition temperature, they form percolating clusters. Denoting the set of broken bonds as *{S_B_}*, we see that ${dim}_{H}\left|S_{B}\right|=0$ at *T* < *T*_g_ and ${dim}_{H}\left|S_{B}\right|=2.5$ at T > *T*_g_, where *dim_H_* denotes the HB dimension of the set of configurons {*S_B_*}, coinciding in our case with the well-known Minkowski box-counting dimension of the set. Configurons, which are bound to their locations in glasses, cannot freely move except by changing their location by thermal hopping through the glass network (disordered lattice of bonds [7]), but they become mobile above the *T*_g_, freely moving via percolating clusters made up of configurons, as any site within it is equivalent for their location. Hence, the configurons acquire new degrees of freedom on passing the *T*_g_. Finally, on transition from the melt to the gaseous phase at higher temperatures ${dim}_{H}\left|S_{B}\right|=3$, that is, the dimension of space available for configuron motion equalises with the dimension of 3D space with all degrees of freedom acquired. The dimensionality of space is connected with conservation of momentum through the concept of translational symmetry, which implies that the physical laws governing the system remain unchanged everywhere in space on moving the system from one location to another. Noether’s theorem formalises this idea by stating that every continuous symmetry of a physical system’s action corresponds to a conservation law [129,130,131]. For translational symmetry, this conservation law is the conservation of momentum. If we consider the configuron moving through the space of percolation clusters, its momentum remains conserved because the percolation cluster space itself does not impose any preferred locations or directions within it, which is true in any number of spatial dimensions. The condition for the transformation of a solid into a liquid and vice versa (when crystallisation or vitrification occurs [132,133]) can be mathematically formulated based on these observations as follows. Namely, one can add a melting criterion based on *set theory,* which, for 3D materials, can be formally based on the following equation:

(5) $${dim}_{H}\left|S_{B}\right|\geq 2.5$$

The fractal dimension *D_H_* of a percolation cluster is always smaller than the dimension *d* of the ambient space, due to numerous “holes” in the cluster. In two dimensions, *D_H_* = 91/48 = 1.90; for d =3, *D_H_* =2.5 [51]. The condition (5) also follows from the Kantor–Webman theorem, which states that the rigidity threshold is identical to the geometrical threshold, and on the equivalence of the elasticity of random percolating networks to regular bond percolation systems [10]. To prove that Kantor and Webman have used the framework of the Born model for the microscopic elasticity of a lattice with elasticity energy given by Hamiltonian: $H_{e}=\frac{1}{2}\sum_{\begin{array}{c} i,j\\ nn \end{array}}K_{i,j}\left[\alpha {\left(u_{i}-u_{j}\right)}_{∥}^{2}+\beta \alpha {\left(u_{i}-u_{j}\right)}_{⊥}^{2}\right]$ with *nn* denoting the nearest neighbours, ${\left(u_{i}-u_{j}\right)}_{∥}$ giving the relative displacement of the site j in the direction parallel to the bond (ij), ${\left(u_{i}-u_{j}\right)}_{⊥}$ giving the relative displacement in the perpendicular direction, and *K_i,j_* being a random variable which assumes values 1 and 0 with probabilities *p* and (1-*p*), respectively. Kantor and Webman found that the rigidity threshold is identical to the geometrical threshold, which allows us to use in practice the well-known properties of percolating clusters; namely, their well-known volume-invariant independence on the type of lattice, valid for disordered lattices of amorphous materials, which, once exceeded, achieves percolation [50,51].

## 6. Importance of Bond Breakage

The distribution of species (atoms, molecules) in amorphous materials looks similar both for glasses, which are solid, and for melts, which are liquid. However, not the species distribution but the degree of connectivity between them determines the state of matter, i.e., the bonding system is crucial so that for more tightly connected atoms, more durable substances occur. The structure of materials is thus presented not solely by atomic distribution but has an integral part in the bonds between them. From this point of view, the structure of glasses is different from the structure of liquids because glasses do not substantially contain broken bonds apart from some remnant defects of temperature-induced breakages, while liquids have a significant part of bonds disrupted, which allows species to exhibit a much higher degree of freedom in motion. Moreover, the structural differences between glasses and melts composed of the same species were noted a long time ago and are readily identified using standard X-ray or neutron scattering techniques based on scattering patterns utilising structure factor *S*(q) or pair distribution functions *g*(r) [2,48,49,52]. Notable is the approach developed by Stoch and Krakowiak [134], who proposed to analyse the temperature changes of radial distribution functions within the whole range of sizes in oxide glasses. In this approach the changes in the further coordination due to other glass constituents are also taken into consideration, which resulted in a better description of the glass–liquid transition and have again confirmed that the transition temperature depends on the glass structure and its thermal changes. Nevertheless, despite this, many works, including university handbooks, focus on just species, affirming that there is no structural difference between a material below and above the glass transition temperature. With an increase of temperature (or other external action on matter, such as pressure and intensity of radiation), chemical bonds between species are broken, and the connectivity gradually decreases until reaching a well-defined threshold level when the bonding system cannot sustain the solid-like behaviour, such as preservation of shape, presence of yield stress for deformation, etc., and the material melts. The transition from solid (vitreous) state to liquid (molten) state is continuous in amorphous materials, and, because of that, the transition is a second-order thermodynamical transition following Ehrenfest classification, although it occurs in a metastable system of topologically disordered species, which, by the total energy, is less favourable compared to an ordered (crystalline) distribution of the same species, which would otherwise minimise it. In contrast with equilibrium phase transitions, which are well understood within statistical mechanics [135,136], the nonequilibrium phase transformations (to which the glass transition undoubtedly belongs) are in reality quite common across many branches of science and technology, ranging from biological systems to cosmology and galactic patterns [137,138,139,140]. The transition from the metastable glass to a stable (or still metastable but with a lower energy) crystalline phase is kinetically impeded, and in many, if not most, natural glasses at room temperature would require times exceeding the lifetime of the universe, which makes these considerations out of any practical sense.

**Figure 6:**
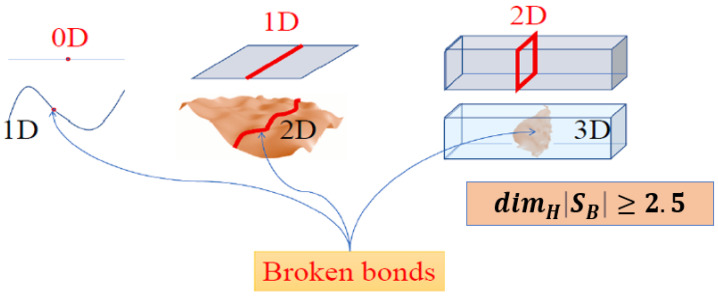
Schematic of “fragmentation” of solids by breaking bonds in the cases when they have 1D (linear), 2D (surface) or 3D (body) dimensionalities of bonds after [2]. Broken bond sets are shown here in red colour and are schematically characterised by 0D (a point), 1D (a line and a curve) or 2D (a plane or a curved surface) dimensionalities. In the case of a 3D amorphous solid material (glass), the structure formed out of configurons at the glass transition temperature is a percolation cluster (a macroscopic fractal structure) which has a dimensionality exceeding 2.5.

Consideration of bonding systems equally with species (atomic and molecular systems) became routine after Angell introduced the concept of configurons as elementary excitations in condensed matter formed by breaking a chemical bond, followed by the associated strain-releasing local adjustment of centres of atomic vibration. In contrast to strongly interacting species, which constitute the matter, the configurons can be, in the first approximation, considered as non-interacting and subject to ideal mixing. In such an approximation, the well-developed apparatus of two-level systems applies, and the glass transition is a percolation effect in the system of configurons—the amorphous material is in the vitreous state until the percolation threshold is reached. The behaviour of percolating systems is universal; thus, it becomes possible to calculate the glass transition temperature, describe diverging heat capacity and CET at glass transition, estimate the constant volume jump of heat capacity, and universally describe the viscosity of material across all temperature intervals from the glass through the melt and gas phases [21,74,75], as well as to understand the kinetic aspect of glass transition [141] and to model it [18,19,20,40,41]. Additionally, it is possible to formalise the melting criterion using the concepts of set theory as the condition of achieving a certain degree of disruption of the bonding system when the HB dimensionality *D_H_* exceeds that of a percolating cluster, which is known for 3D space to be larger or about 2.5 (Figure 6).

Characterising the predictive ability of models, Doremus emphasised that the bonding between molecules, defects, and the structure are much more important than the free volume [142]. In contrast with known free volume or mode coupling models of glass transition [143,144,145], the CPT relies on and operates with the effective volume of chemical bonds and configurons [8,19] rather than with the excess or free volume of the material that is found as the difference between the specific volume of the material and the volume of atoms/molecules involved. It bases its conclusions on a percolation-type phase transformation at the calorimetric glass transition with supporting evidence on phase transformation through observation of massive fluctuations of derivative parameters of the system—see e.g., Ref. [146]. Notably also that in line with CPT conclusions for both physical and computer-based experiments, the results are affected by the size of the system under consideration [18,19,20,21,22,147,148].

## 7. Conclusions

While the calorimetric glass transition in amorphous materials is an obvious effect mechanically expressed by the solid-like behaviour, such as the brittleness of glasses, against liquid-like behaviour, including the plasticity of the molten state, the underlying atomic-size mechanisms and structural rearrangements responsible for the transition itself are still poorly understood. This resulted in widely spread claims that there is no structural difference between glasses and liquids. In the meantime, the viscosity being even used on the logarithmic scale cannot serve as a criterion of glass transition because the viscosity of materials at the calorimetric glass transition temperature spans over four orders of magnitude. Moreover, the treatment of vitrification as a process of continuously breaking ergodicity with entropy loss and a residual entropy tending to zero in the limit of zero absolute temperature disagrees with most experimental and theoretical investigations. Structural rearrangements at calorimetric glass transition are behind drastic changes of material characteristics, causing differences between glasses and melts. The structural description of materials includes both species (atoms, molecules) and connecting bonds, which are directly affected by changing conditions such as the increase of temperature when the bonding system becomes softer. At and above the glass transition a macroscopic percolation cluster made up of configurons (broken chemical bonds) is formed which causes changes in the physical properties of the material from those of a solid to those of a liquid. An account of a percolation cluster made out of configurons enables the structural differentiation of glasses from melts. Here, we have highlighted the role of the bonding system in understanding the nature of the transformation of glasses into melts. Considering glass–liquid transition as the melting of amorphous solids, we formulated the melting criterion of solids as the condition of increase of Hausdorff–Besicovitch dimensionality of the set of configurons from zero to 2.5.

## Figures and Tables

**Table 1:** Examples of materials with different types of bonding and the corresponding configurons.

Bond Type	Substance	Bond Energy (kJ/mol)	Configuron Description	Microscopic Result of Configuron Formation
Covalent	SiO_2_	443	A Si–O broken bond with neighbouring adjustments	A shift by one or more atoms from the first coordination shell
Ionic	CuF_2_	2591	A Cu–F broken bond with neighbouring adjustments	Same as above
Metallic	Fe	407	A displacement of an atom out of the first coordination shell with neighbouring adjustments	Same as above
Van der Waals	Ar	7.6	A broken Ar–Ar bond with neighbouring adjustments	Same as above
Hydrogen	H_2_O	50	A broken hydrogen bond with neighbouring adjustments	Same as above

**Table 2:** Phases of materials as a function of connectivity and ordering of atomic constituents.

Degree of Ordering	Degree of Connectivity	
	Low	High
High	Liquid crystals; Liquid quasi-crystals	Crystals; Quasi-crystals
Medium	Liquid glasses	Glass–crystalline materials
Low	Melts	Glass ^1^

^1^ The connectivity between atomic species can be diminished not only by an increase of temperature: the irradiation of glasses, which breaks the interatomic chemical bonds, leads to fluidisation of glasses [9,43,44,45].

**Table 3:** Viscous flow types and the CPT universal viscosity equation, also known as the DDO model [73,74].

Table 1.	Low (in the Glass) *T < T_g_*	Intermediate (in the Supercooled Melt) *T_g_* < *T* < *T_A_*	High (in the Melt) *T* > *T_A_ =* (1.10 ± 0.15) *T_m_*	Extremely High
Viscous flow type	Arrhenian with high activation energy Q_H_	Non-Arrhenian, apparent variable activation energy Q(T)	Arrhenian with low activation energy Q_L_	Non-activated, growing with temperature rise
CPT universal viscosity equation ^1^	$\eta \left(T\right)=A_{1}T\left[1+A_{2}\exp\left(\frac{Q_{L}}{RT}\right)\right]\left[1+C\exp\left(\frac{Q_{H}-Q_{L\;}}{RT}\right)\right]$			

^1^ The universal viscosity equation resulting from CPT correctly predicts the minima of viscosities at very high temperatures [75]. The DDO viscosity model based on CPT is also supported by experimental data on the transition of flow of vitreous materials to a low activation mode under electron irradiation [9,43,44,45].

**Table 4:** Comparison of some constant volume heat capacity jumps at calorimetric glass transition with CPT value ∆Cv = 10.4–10.8 J/mol·K.

Alloy, Compound	*T_g_*, K	*∆C_v_*, J/mol·K
La_55_Al_25_Ni_20_	465	12.31
Zr_65_Al_7.5_Ni_10_Cu_17.5_	653	11.02
Mg_65_Cu_25_Y_10_	380	10.06
Zr_41.2_Ti_13.8_Cu_12.5_Ni_10_Be_22.5_	623	11.95
Pd_77.5_Cu_6_Si_16.5_	625	10.33
Pd_40_Cu_30_Ni_10_P_20_	525	10.89
Pd_40_Ni_40_P_20_	551	11.02
Zr_55_Al_10_Ni_5_Cu_30_	653	11.32

## Data Availability

No new data were created or analyzed in this study. Data sharing is not applicable to this article.

## Abbreviations

The following abbreviations are used in this manuscript:

CET	Coefficient of thermal expansion
CPT	Configuron percolation theory
DSC	Differential scanning calorimetry
HB	Hausdorff–Besicovitch dimensionality
MRO	Medium-range order
SRO	Short-range order
